# A Munificent Gift

**Published:** 1894-01-27

**Authors:** 


### A MUNIFICENT GIFT.

We have received the following letter from the Secretary of the Edinburgh Royal Hospital for Sick Children: "We have to direct your attention to the fact that your paragraph in The Hospital of 20th inst. understates the munificent donation to the new hospital by Lady Jane Dundas. Besides the donation of £6,500 for the building of a wing of the new hospital, she gave £5,000 to be applied to the maintenance of the hospital in the endowment of beds in that wing. We should much regret that anyone should be under a misapprehension as to her ladyship's generosity."

